# Signals from intestinal microbiota mediate the crosstalk between the lung-gut axis in an influenza infection mouse model

**DOI:** 10.3389/fimmu.2024.1435180

**Published:** 2024-07-23

**Authors:** Yijia Zhang, Youdi Wan, Xin Xin, Yixuan Qiao, Wenna Qiao, Jihui Ping, Juan Su

**Affiliations:** ^1^ Laboratory of Animal Neurobiology, College of Veterinary Medicine, Nanjing Agricultural University, Nanjing, China; ^2^ Ministry of Education (MOE) Joint International Research Laboratory of Animal Health and Food Safety, Engineering Laboratory of Animal Immunity of Jiangsu Province, College of Veterinary Medicine, Nanjing Agricultural University, Nanjing, China

**Keywords:** influenza A virus, gut-lung axis, intestinal microbiota, tryptophan metabolism, *Lactobacillus*

## Abstract

**Introduction:**

Introduction: The influenza virus primarily targets the respiratory tract, yet both the respiratory and intestinal systems suffer damage during infection. The connection between lung and intestinal damage remains unclear.

**Methods:**

Our experiment employs 16S rRNA technology and Liquid Chromatography-Mass Spectrometry (LC-MS) to detect the impact of influenza virus infection on the fecal content and metabolites in mice. Additionally, it investigates the effect of influenza virus infection on intestinal damage and its underlying mechanisms through HE staining, Western blot, Q-PCR, and flow cytometry.

**Results:**

Our study found that influenza virus infection caused significant damage to both the lungs and intestines, with the virus detected exclusively in the lungs. Antibiotic treatment worsened the severity of lung and intestinal damage. Moreover, mRNA levels of *Toll-like receptor 7* (*TLR7*) and *Interferon-b* (*IFN-b*) significantly increased in the lungs post-infection. Analysis of intestinal microbiota revealed notable shifts in composition after influenza infection, including increased *Enterobacteriaceae* and decreased *Lactobacillaceae*. Conversely, antibiotic treatment reduced microbial diversity, notably affecting *Firmicutes*, *Proteobacteria*, and *Bacteroidetes*. Metabolomics showed altered amino acid metabolism pathways due to influenza infection and antibiotics. Abnormal expression of indoleamine 2,3-dioxygenase 1 (IDO1) in the colon disrupted the balance between helper T17 cells (Th17) and regulatory T cells (Treg cells) in the intestine. Mice infected with the influenza virus and supplemented with tryptophan and *Lactobacillus* showed reduced lung and intestinal damage, decreased *Enterobacteriaceae* levels in the intestine, and decreased IDO1 activity.

**Discussion:**

Overall, influenza infection caused damage to lung and intestinal tissues, disrupted intestinal microbiota and metabolites, and affected Th17/Treg balance. Antibiotic treatment exacerbated these effects. Supplementation with tryptophan and *Lactobacillus* improved lung and intestinal health, highlighting a new understanding of the lung-intestine connection in influenza-induced intestinal disease.

## 1 Introduction

Due to the high levels of morbidity and mortality caused by the influenza virus worldwide, it is urgent to explore effective means to prevent and treat it. Respiratory symptoms are the major clinical manifestations of influenza infection, usually accompanied by gastrointestinal reactions. Understanding the underlying mechanisms that mediate the interaction between the lung and gastrointestinal tract has become a topic of great interest. Recent studies have clarified the importance of regulating intestinal microorganisms in host immunity (1, 2). The mechanisms by which the influenza virus alters intestinal microbiota are not fully understood, although several potential pathways may be involved. Work in our laboratory and others have demonstrated that genes associated with the Toll-like receptors (TLRs) signaling pathway and the downstream cytokines such as type I interferons (IFN-Is) play a key role in the alteration of intestinal microbiota induced by influenza virus infection. Toll-like receptors are a family of pattern recognition receptors (PRRs) in the innate immune response (3). TLRs can specifically recognize pathogen-associated molecular patterns (PAMPs) and transmit pathogen-related molecular signals into cells (4). Multistep signaling cascades can be initiated and subsequently trigger adaptive immune responses. Toll-like receptor-mediated IFN-I induction plays an important role in facilitating antiviral responses (5). Type I IFNs produced in the lungs, which are central to antiviral defenses, enhanced the inhibitory effect on the intestinal antibacterial and inflammatory responses (6).

In the intestine, commensal microbes assist in providing the body with sufficient and essential nutritional products (7). Therefore, the disturbance of microbial balance might have substantial consequences on host health through host microbial metabolism (8) and T cell response (9). Dysbacteria is often characterized by a depletion of obligate anaerobic bacteria and an enrichment of *Proteobacteria* in the gut (9, 10). In our study, when the delicate balance of intestinal microbiota was disrupted by the influenza virus and antibiotics, the number of *Enterobacteriaceae* increased while *Lactobacillaceae* decreased. It has become increasingly apparent that the microbiota modulates signaling pathways contributing to mucosal immune homeostasis through metabolites. The effects of several specific microbiota-derived metabolites on the immune system have been identified, including short-chain fatty acids (11, 12), indole (13), and polysaccharide A (14, 15). Among the microbial metabolites in the intestines, we are particularly interested in the tryptophan metabolism pathway. As an essential amino acid in mammals, tryptophan metabolites have been shown to be involved in gut immune homeostasis by regulating regulatory T cells (Tregs) and helper T17 cell (Th17) responses mediated by indoleamine 2,3-dioxygenase 1 (IDO1) (16, 17). Disruption of the balance between Th17 and Treg can induce immune injury in the intestine, with antibiotic treatment exacerbating such damage.

Thus, our study has revealed an interaction between the lungs and intestines mediated by commensal microbiota, which captures signals from microbial metabolites and subsequently alter the gastrointestinal microenvironment, exacerbating or alleviating intestinal injury.

## 2 Materials and methods

### 2.1 Mice and virus

Female BALB/c mice, with an average weight of (20 ± 2) g and 6–7 weeks old, were purchased from Shanghai Model Organisms Center and housed under SPF conditions. All animals were housed for 7 days to adjust to housing conditions under a strict 12-h light/dark cycle. In total, 50 mice were randomly divided into the mock-infected group, the virus-infected group, the antibiotic treatment group, the *Lactobacillus* treatment group, and the tryptophan treatment group (10 each). A/WSN/33 (H1N1; WSN) influenza virus was propagated in the allantoic cavity of 9-day-old embryonated chicken eggs. Titers were determined by a standard plaque assay on MDCK cells and viral stock was stored at -80°C. Mice were fully anesthetized by inhalation of diethyl ether and then infected with a sublethal dose (200 PFU) of the mouse-adapted A/WSN/33 (H1N1; WSN) influenza virus strain or Phosphate-Buffered Saline (PBS) by non-surgical intratracheal instillation.

### 2.2 Antibiotics, *Lactobacillus*, and tryptophan treatments

For the antibiotic treatment group, 1 day after infection with the virus, mice were treated with ampicillin (1 g/L), vancomycin (500 mg/L), neomycin sulfate (1 g/L), and metronidazole (1 g/L) by drinking water as previously described (18). For the *Lactobacillus* and tryptophan treatment groups, 1 week before infection with the virus, mice were treated with *Lactobacillus* (10^8^ CFU/mL) and tryptophan (100 mg/kg/bw) by intragastric administration. Mice were monitored daily and weighed until day 9, after which all the mice were euthanized. Animal care and experimental procedures were followed in accordance with the experimental animal guidelines at USTC.

### 2.3 Determination of virus

Influenza virus in the lungs, colons, and small intestines was detected by PCR. The primer sequences to detect the gene encoding the matrix protein within the influenza virus were as follows: 5’ -GGACTGCAGCGTAGACGCTT-3’ (forward) and 5’ -CATCCT- GTTGTATATGAGGCCCAT-3’ (reverse).

### 2.4 Histopathology

Lung, colon, and small intestine tissues were collected and fixed immediately in 4% neutral-buffered formalin in PBS for>24 h, embedded in paraffin, and cut into 5–7µm sections. The sections were deparaffinized and stained with hematoxylin and eosin (H&E) to observe histological changes. Generic grading criteria histopathologic grades of the lungs were assigned as grade 0 (none), grade 1 (mild), grade 2 (moderate), and grade 3 (severe) based on an increasing extent and/or complexity of change (**Table 1**) (19–21). Generic grading criteria histopathologic grades of the intestine were assigned as grade 0 (none), grade 1 (mild), grade 2 (moderate), and grade 3 (severe) based on an increasing extent and/or complexity of change (**Table 2**) (22, 23).

**Table 1:** Histopathologic grades of the lung.

	Grade 0	Grade 1	Grade 2	Grade 3
Apoptotic bodies in bronchi walls	None	Mild	Moderate	Severe
Intra-alveolar hemorrhage	None	Mild	Moderate	Severe
Vasculitis	None	Mild	Moderate	Severe
Peribronchial inflammation	None	Mild	Moderate	Severe
Perivascular inflammation	None	Mild	Moderate	Severe
Bronchopneumonia	None	Mild	Moderate	Severe
Perivascular edema	None	Mild	Moderate	Severe
Parenchymal edema	None	Mild	Moderate	Severe

**Table 2:** Histopathologic grades of the intestine.

	Grade 0	Grade 1	Grade 2	Grade 3
Inflammatory cell infiltration	No inflammatory cell infiltration	Mild inflammatory cell infiltration	Moderate inflammatory cell infiltration	Severe inflammatory cell infiltration
Crypt atrophy	No atrophy	Mild atrophy	Moderate atrophy	Severe atrophy
Crypt damage	No damage	Mild damage	Moderate damage	Severe damage
Epithelial damage	No damage	Mild damage	Moderate damage	Severe damage
Ulceration	No ulcer	Mild ulcer	Moderate ulcer	Severe ulcer

### 2.5 Real-time PCR

Total RNA was extracted from lung, colon, and intestine samples with the RNA isolator (Vazyme). The concentration and quality of the RNA were measured with a Nano Drop ND-1000 Spectrophotometer (Thermo Fisher Scientific, Madison, WI, USA). Next, 2 μg of total RNA was treated with RNase-Free DNase (M6101; Promega, Madison, WI, USA) and was reverse transcribed according to the manufacturer’s instructions using a SYBR Premix Ex *Taq* (*Vazyme*). Two microliters of diluted cDNA (1:20, vol/vol) were used for real-time PCR with Mx3000 P Real-Time PCR System (Stratagene, USA). For analysis, the expression of the target gene was normalized to the housekeeping gene β-actin. Values of gene expression were then calculated using the mean from the control samples as a calibrator. The relative mRNA level of the target gene in each treatment group = the mRNA level of the target gene in the treatment group/mRNA level of the target gene in the control group. Primers were synthesized by Generay Biotech. A list of qPCR primers used in this study is provided in **Table 3**.

**Table 3:** Sequences of primers used for RT-PCR.

Gene	Primer Sequences (5′–3′)
*Virus matrix protein*	F: GGACTGCAGCGTAGACGCTT R: CATCCTGTTGTATATGAGGCCCAT
*TLR7*	F: CTTTGGACCCCAGTAGAACAG R: GCACAGACAAGCATTTGACAG
*Myd88*	F: ACGGTCGGACACACACAAC R: TCCCACAAACAAAGGAACTG
*IFN-α*	F: AAGAGGTTCAAAATCTGCTGG R: AAGAAATGAGAAGACTCCCCC
*IFN-β*	F: CATCTTCTCCGTCATCTCCATA R: TGCGTTCCTGCTGTGCTT
*IDO1*	F: AGAGGATGCGTGACTTTGTG R: ATACAGCAGACCTTCTGGCA
*RORγ*	F: CCGCTGAGAGGGCTTCAC R: TGCAGGAGTAGGCCACATTACA
*Foxp3*	F: GGCAATAGTTCCTTCCCAGAGTT R: GGGTCGCATATTGTGGTACTTG
*GAPDH*	F: CCTCGTCCCGTAGACAAAATG R: TCTCCACTTTGCCACTGCAA
*All bacteria*	F: ACTCCTACGGGAGGCAGCAGT R: ATTACCGCGGCTGCTGGC
*Enterobacteriaceae*	F: GTGCCAGCMGCCGCGGTAA R: GCCTCAAGGGCACAACCTCCAAG
*Lactobacillus*	F: AGCAGTAGGGAATCTTCCA R: CACCGCTACACATGGAG

F, forward primer; R, reverse primer.

### 2.6 Bacteria preparation

*Lactobacillus brevis* was provided by Shanghai Fuxiang Biotechnology Co., LTD, incubated in MRS broth (DeMan, Rogosa, Sharpe) under anaerobic conditions at 37 °C for 48 h. The optical density (OD) was measured at 620 nm to adjust the final concentration of the bacterial suspension, and the exact number of CFU was determined by plating serial dilutions of the inoculum onto MRS.

### 2.7 Analysis of the fecal microbiota

Fecal samples were collected from groups at 9 dpi, and then snap frozen in liquid nitrogen. In total, 40 mg of fecal sample from each mouse was subjected to DNA extraction according to a bead-beating method and followed by phenol-chloroform extraction (24). DNA extracts were diluted in 50 μl ddH_2_O prior to PCR reactions. Furthermore, 2 μl of extracted fecal bacterial DNA was used as a template for 16S qPCR reaction, operational taxonomic units (OTU) were identified and the linear discriminant analysis effect size (LEfSE) analysis was performed by Beijing Genomics Institute (BGI).

### 2.8 Analysis of the microbial metabolites

Fecal samples were first derivatized according to published methods with few modifications and analyzed with Gas Chromatography/Time-of-Flight Mass Spectrometry (GC/TOF-MS) (25). After a derivative process on a 10 mg fecal sample from each mouse was performed, 1 μL of the derivatized solution was injected into an Agilent 6890N gas chromatograph that was coupled with a Pegasus HT time-of-flight mass spectrometer (GC/TOFMS, Leco Corp., Joseph, MI) in splitless mode at 260˚C. Chroma TOF software v4.22 (Leco) was used for the multivariate analysis after the data from the GC/TOF-MS mass spectrogram and chromatogram were pretreated. All of the procedures were conducted according to previous publications (26). Metabolite identification was performed with SIMCA-p (13.0) software using PLS-DA and PCA models and metabolic pathways analysis was performed using Metaboanalyst software. The concentration of metabolites in the tryptophan pathway of the *Lactobacillus* and tryptophan treatment groups was analyzed with Liquid Chromatography-Mass Spectrometry (LC-MS). Thus, a 50 mg sample was placed into 2 mL centrifuge tube, 100 mg glass beads was added along with 200 μL 10% methanol formate solution and 200 μL sterile water and ground for 60 s in 40 HZ, and repeated two times. After centrifugation at 12000 rpm at 4°C for 10 min, 10 μL of the supernatant was diluted at a rate of 50 times as the high-content compound to be tested, and the supernatant was taken as the low-content compound to be tested. This was done using a C18 chromatographic column (2.1×100 mm, 1.7 m, Waters, USA) with an injection volume of 5 μL and a column temperature 40°C. Mobile phase A was 10% methanol (0.1% formic acid) and B was 50% methanol (0.1% formic acid). Gradient elution was performed and the flow rate was 0.4 mL/min. The mass spectrometer utilizes an electrospray ionization (ESI) source in positive ion ionization mode to detect intestinal metabolites. The ion source temperature was 500°C, the ion source voltage was 5500 V, the collision gas was 6 psi, the curtain gas was 30 psi, and the atomizing gas and the auxiliary gas were both 50 psi. Scanning was performed using multiple response monitoring (MRM).

### 2.9 Western blot

Total protein was extracted from mouse colon and intestine tissues using RIPA cracking liquid with PMSF inhibitors. In total, 40 μg of total protein was resolved using 12% SDS-PAGE gels and transferred to PVDF membranes. The membranes were blocked with 4% nonfat dried milk and incubated at 4°C with primary antibodies. Detection of mouse GAPDH was performed with primary mouse monoclonal antibody (MultiSciences Biotech), while detection of Indoleamine 2,3-dioxygenase 1 (IDO1) was performed with monoclonal mouse antibody (Proteintech). Retinoic acid-related orphan receptor gamma (RORγ) was detected by monoclonal rat antibody (Invitrogen). Forkhead box p3 (Foxp3) was detected by polyclonal rabbit antibody (Proteintech). After overnight incubation at 4°C, the blots were washed and then incubated for 2 h at room temperature with secondary goat anti-mouse, goat anti-rat, and goat anti-rabbit antibodies conjugate to horseradish peroxidase (HRP) (MultiSciences Biotech and Invitrogen, respectively). Then, the membranes were washed with TBST for 30 min, followed by detection with chemiluminescence and image densitometry analysis with a Tanon-6200 luminescent image analysis system (Tanon-6200, China) with BeyoECL Plus (Beyotime Biotechnology, China). After washing, bands were developed using followed by detection with chemiluminescence and image densitometry analysis with a Tanon-6200 luminescent image analysis system (Tanon-6200, China) with BeyoECL Plus (Beyotime Biotechnology, China).

### 2.10 Flow cytometry

The ileum and colon were longitudinally sliced into sections measuring 0.5 cm and then rinsed in Hanks’ Balanced Salt Solution containing 2% FCS. The tissues were placed in HBSS with 2 mM EDTA at 37°C for 20 minutes in a shaking incubator, incubated twice. Subsequently, they were digested in complete RPMI with 1 mg/mL Collagenase VIII (Sigma-Aldrich) at 37°C for 15 minutes, or until complete digestion of the tissue occurred. Lymphocyte separation was performed by using a Percoll lymphatic separator kit (Solarbio, Beijing, China). For surface markers, cells were incubated with fluorochrome conjugated antibodies against surface markers at 4°C for 30 min in the dark. For intracellular markers, cells were fixed and permeabilized with a Cytofix/Cytoperm TM Fixation/Permeabilization Solution Kit (BD Biosciences, catalog 554714) or a Foxp3/Transcription Factor Staining Buffer Set (eBioscience, catalog 00–5523-00), and then incubated with fluorochrome-conjugated antibodies at 4°C for an additional 30 min. Flow cytometric analyses utilized antibodies (Abs) that were acquired from BD Biosciences. Prior to staining, the cells underwent pre-treatment using Mouse BD Fc Block (BD Pharmingen, Franklin Lakes, NJ). Subsequently, they were stained with FITC-conjugated anti-CD4, PE-conjugated anti-RORγt, and APC-conjugated anti-Foxp3 following the guidelines provided by the manufacturer (Treg cells: CD4+Foxp3+; Th17 cells:CD4+Foxp3-RORγt+). At the same time, cells also made relevant isotype controls. Cell frequencies were calculated by adding Spherotech Accucount blank particles. Flow cytometry was performed on a FACS Calibur analyzer using the FACS Diva 6.2 software (BD Biosciences). Subsequently, the data was analyzed with FlowJo software.

### 2.11 Statistical analysis

The values of each group were shown as the mean ± standard error of the mean (SEM). Before analyses, we used the Kolmogorov–Smirnov test to assess whether the data were normally distributed. The variables met the assumptions for normality except for the sequencing data. Then, we used the independent samples T test to evaluate the significant difference between two different treatment groups by using SPSS 25.0 software (SPSS, Chicago, IL, USA). Correlation analysis, expressed as Spearman’s correlations (R > 0.5, P < 0.05), was performed to determine the correlations between the abundance of microbiota and intestinal development.

## 3 Results

### 3.1 Crosstalk between lung and intestines after influenza virus infection

**Figure 1:**
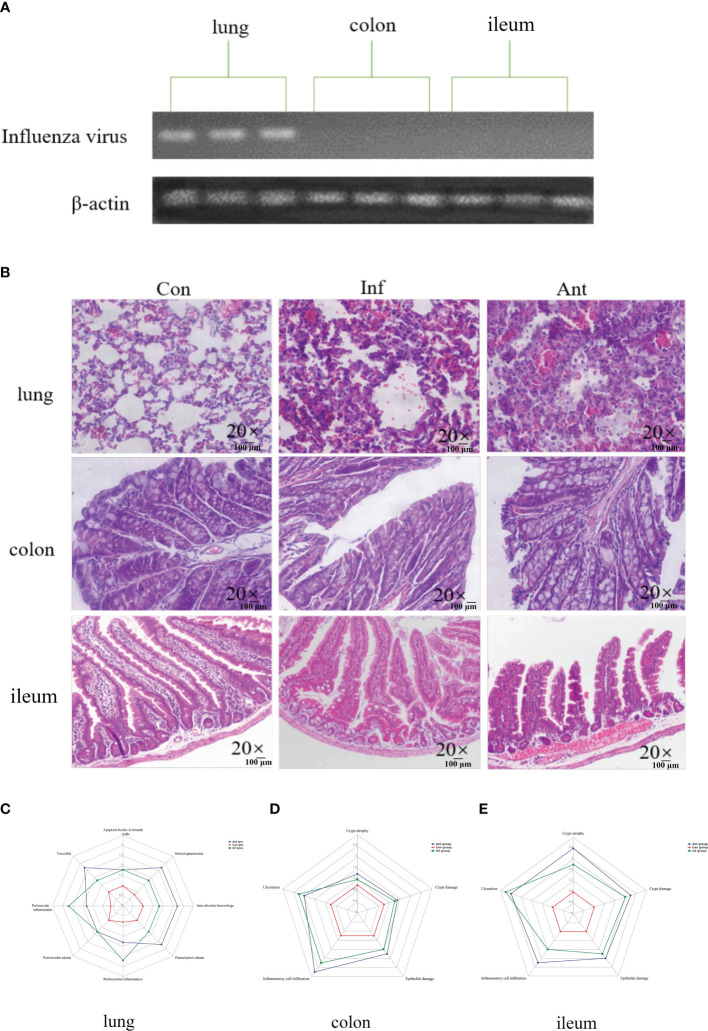
Influenza virus infection causes lung and intestinal immune injury. **(A)** The levels of the influenza virus–derived matrix protein gene in the lungs, colon, and ileum were detected by PCR; **(B)** Histology analysis including H&E (scale: 100 μm) of the lungs, colon, and ileum was observed at 9 days post infection; **(C)** Spider web plot displaying histopathological scoring of lung damage; **(D)** Spider web plot displaying histopathological scoring of colon damage; **(E)** Spider web plot displaying histopathological scoring of ileum damage. Con: Female BALB/c mice aged 6 to 7 weeks (20 ± 2 g) were treated with PBS; Inf group: Female BALB/c mice aged 6 to 7 weeks (20 ± 2 g) were treated with influenza virus infection; Ant group: Female BALB/c mice aged 6 to 7 weeks (20 ± 2 g) were treated with a combination of antibiotics.

In this study, mice were subjected to the H1N1 influenza virus to establish a mouse model of respiratory influenza infection. To confirm that the animal model was successfully established, we detected the expression of the influenza virus-derived matrix protein gene in the lungs, colon, and ileum at 9 days post infection (**Figure 1A**). In line with a previous study, we could only detect the presence of the virus in the lungs, revealing that respiratory epithelial is the primary target of influenza virus replication, and no infectious particles were found in the colon and ileum. The mice were then treated with combinatorial antibiotics via drinking water to perturb the intestinal microbiota 1 day after infecting them with H1N1 influenza virus. In addition to respiratory clinical signs, virus-and antibiotic-treated mice displayed gastrointestinal tract symptoms, including decreased food appetite, wet feces, and significant weight loss compared with the mock-infected mice. We observed the histopathology of the lung and intestine tissues at 9 dpi (**Figure 1B**). As expected, there were varying degrees of damage in the lungs and intestines of mice treated with the virus and antibiotics (**Figures 1C–E**). Histological analyses showed that inflammatory cell infiltration and pulmonary interstitial hyperplasia were evident in the lungs (**Figure 1C**) and the intestine tissues (**Figures 1D, E**) were disrupted in both groups. However, the injury in the intestines of the antibiotic group was more serious than that in the virus-infected group. These data indicated that immune injury in the intestines is indirectly caused by the influenza virus, and there may be underlying mechanisms connecting the lungs with the intestines.

### 3.2 TLR7 signaling and *IFN-α* and *IFN-β* mRNA expression in the lungs and their correlation with *viral RNA* mRNA expression

**Figure 2:**
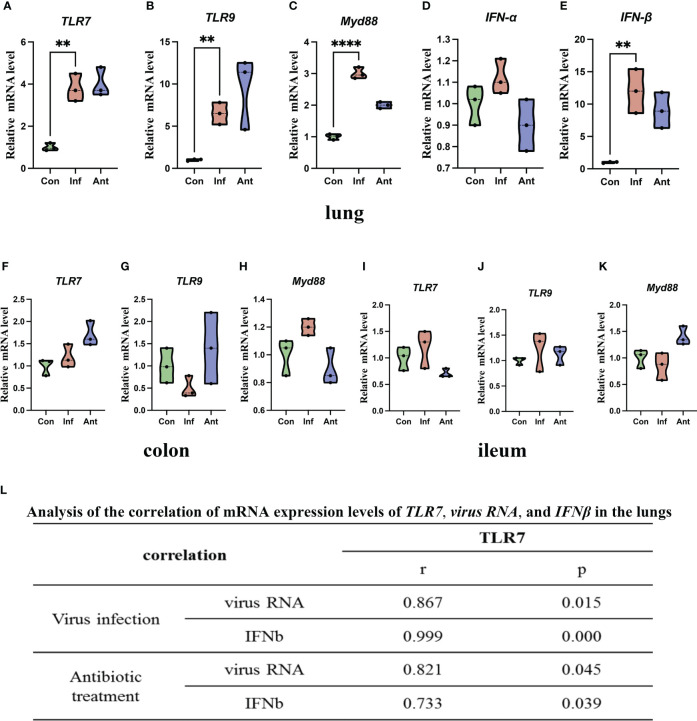
Increased viral RNA, TLR7 signaling, and IFN-α and IFN-β mRNA expression in the lungs, colon, ileum, and their correlation. **(A)** TLR7 mRNA level; **(B)** TLR9 mRNA level; **(C)** Myd88 mRNA level; **(D)** IFN-α mRNA level; **(E)** IFN-β mRNA level in the lungs and **(F)** TLR7 mRNA level; **(G)** TLR9 mRNA level; **(H)** Myd88 mRNA level in the colon; **(I)** TLR7 mRNA level; **(J)** TLR9 mRNA level; **(K)** Myd88 mRNA level in the ileum. **(L)** Expression of TLR7 correlated with viral RNA and IFN-β mRNA levels in the lungs of the virus- and antibiotic-treated mice. Each dot represents data from one animal. Data are mean ± SEM. n = 3. Con: Female BALB/c mice aged 6 to 7 weeks (20 ± 2 g) were treated with PBS; Inf group: Female BALB/c mice aged 6 to 7 weeks (20 ± 2 g) were treated with influenza virus infection; Ant group: Female BALB/c mice aged 6 to 7 weeks (20 ± 2 g) were treated with combination of antibiotics. Statistical analysis was performed by independent samples T test. **P < 0.01; ****P < 0.0001. All replicates are biological.

TLRs play an important role in host defense against pathogens, and Toll-like receptor 7 (TLR7) can recognize the ssRNA of the influenza virus (27). Furthermore, TLR7 signaling also induces the production of downstream cytokines, such as IFN-α and IFN-β, which play a key role in facilitating antiviral responses (28). Base on the above, we hypothesized that TLR7 signaling may mediate the antiviral effect through the production of IFN-α and IFN-β during the influenza virus infection. To explore this hypothesis, we tested the mRNA levels of *virus RNA*, *TLR7*, *Myeloid differentiation primary response 88* (*MYD88*), *Interferon-alpha* (*IFN-α*), and *Interferon-gamma* (*IFN-β*) in the lungs (**Figures 2A–E**). *Virus RNA*, *TLR7*, *MYD88*, and *IFN-β* mRNA levels increased significantly in the lungs of virus-infected and antibiotic-treated mice compared with the mock-infected mice. However, there was no significant difference in *IFN-α* mRNA level in the lungs between the experimental groups and the control group, which suggested that the influenza virus infection did not induce change in IFN-α in the lungs. In addition, we also detected the mRNA levels of *TLR7*, *TLR9*, and *MYD88* in the colon (**Figures 2F–H**) and ileum (**Figures 2I–K**). Q-PCR results showed that there were no significant differences among the three groups in the mRNA levels of *TLR7*, *TLR*9, and *MYD88* in both the colon and ileum. To assess the relationship between *TLR7* and *virus*, *IFN-β* mRNA expression in the lungs of virus-infected and antibiotic-treated groups, we investigated the correlation of *TLR7* mRNA level with *viral RNA* and *IFN-β* mRNA level and found that *TLR7* mRNA level directly correlated with *viral RNA* and *IFN-β* mRNA level (**Figure 2L**). These findings collectively suggested that the upregulation of TLR7 in the lungs of virus-infected and antibiotic-treated mice is associated with influenza virus replication and IFN-β induction.

### 3.3 Antibiotics exacerbate the alteration of the intestinal microbiota

**Figure 3:**
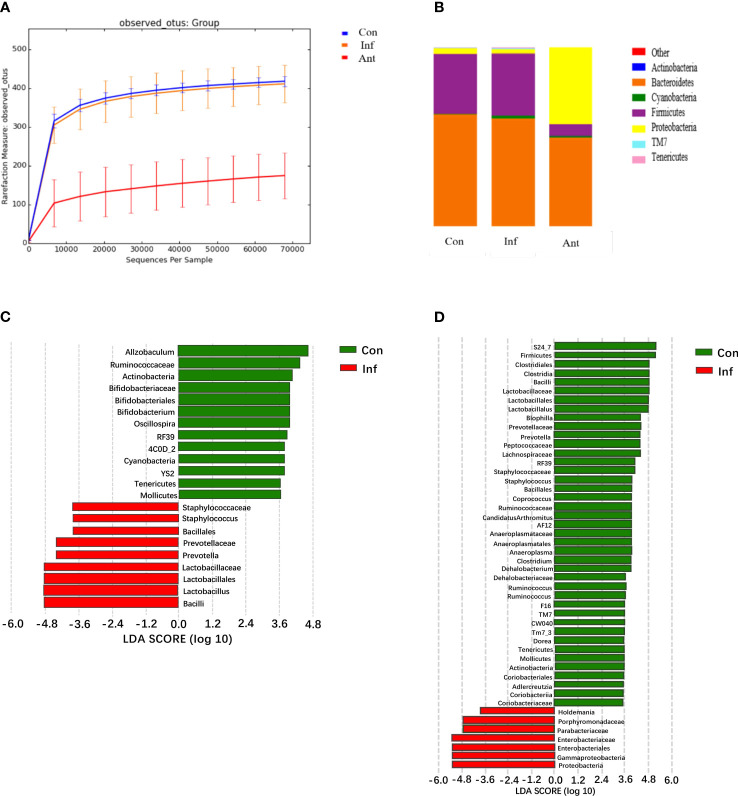
H1N1 influenza virus-induced IFN-β alters the fecal microbiota diversity and composition; analysis of the fecal microbiota from the control, virus-infected mice, and antibiotic-treated mice during influenza infection. **(A)** The diversity of fecal microbiota from experimental groups on the 9th day post infection (dpi) was analyzed by sequencing; **(B)** Graph is the average relative abundance of each bacterial phylum; **(C, D)** LDA score about control and virus infection group, control, and antibiotic-treated group. Con: Female BALB/c mice aged 6 to 7 weeks (20 ± 2 g) were treated with PBS; Inf group: Female BALB/c mice aged 6 to 7 weeks (20 ± 2 g) were treated with influenza virus infection; Ant group: Female BALB/c mice aged 6 to 7 weeks (20 ± 2 g) were treated with combination of antibiotics.

Previously, it was reported that IFN-α and IFN-β were induced in the lungs during influenza pulmonary infection and this altered the intestinal microbiota. In our research, it was clear that IFN-β might provide important signals critical for shaping the composition of the intestinal microbiota. Therefore, we analyzed the microbiota diversity and composition in the fecal contents of each group on the 9th day post infection (**Figures 3A, B**). Unexpectedly, our data showed that the intestinal microbiota had subtle changes in diversity and composition, but without alteration of bacterial community richness during influenza infection alone. On the contrary, microbial DNA extracted from fecal samples of the antibiotic-treated mice showed that antibiotic treatment could drastically alter the richness and components of the intestinal microbiota. Intuitively, antibiotic treatment has a more robust impact on microbiota composition. Although bacterial species showed some subtle differences between the virus- and mock-infected mice, *Bacteroidetes* and *Firmicutes* remained the most dominant colonizers, and a low abundance of *Proteobacteria* in the influenza infected group, which was consistent with the composition of normal intestinal microorganisms as previously reported (29, 30).

In contrast to the virus-infected mice, intestinal microbial diversity in the antibiotic-treated mice was significantly decreased compared with the mock-infected mice. Although *Bacteroidetes* and *Firmicutes* were still the major bacterial phyla in the fecal microbial community of antibiotic-treated mice, a significant blooming of *Proteobacteria* was noticed. Additionally, the results displayed a significantly lower level of *Firmicutes* in the stool samples of the antibiotic-treated mice compared with the mock- infected mice, and no statistical differences were found in the level of *Bacteroidetes* between them. To investigate which bacterial species contributed to the differences in intestinal microbiota in the virus-infected and antibiotic-treated mice, we performed linear discriminant analysis effect size (LEfSE) analysis (**Figures 3C, D**). In line with expectations, *Enterobacteriaceae* and *Lactobacillaceae* were involved in the bacterial species alterations in antibiotic-treated mice, as *Enterobacteriaceae* and *Lactobacillaceae* are the most relevant species of *Proteobacteria* and *Firmicutes*. The data also showed a change in the *Lactobacillaceae* of the virus-infected mice. These results indicated that the most striking changes in the fecal microbial community of the antibiotic-treated mice were the increased numbers of *Enterobacteriaceae* and the decreased numbers of *Lactobacillaceae* (31).

### 3.4 Tryptophan metabolism destroys the T cell balance in the intestines

**Figure 4:**
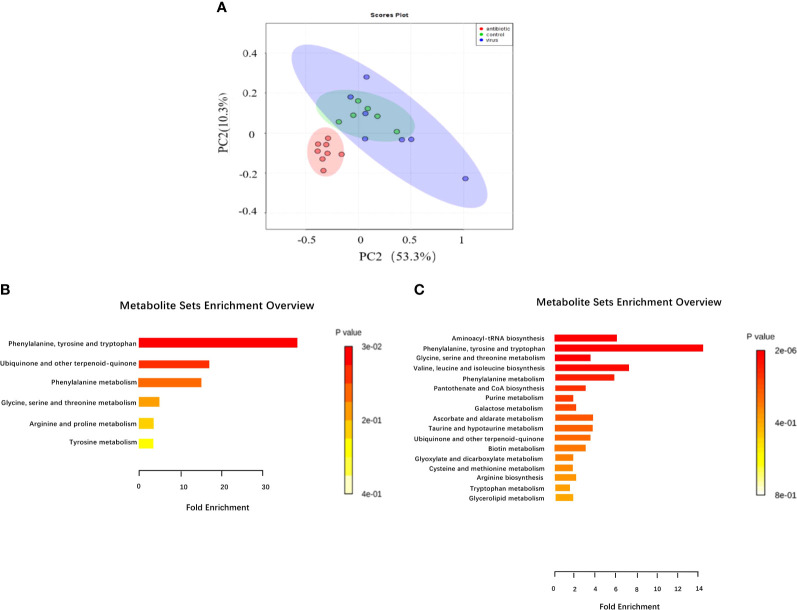
Analysis of fecal metabolic characteristics associated with H1N1 influenza virus infection by GC/TOF-MS. **(A)** Fecal matter from mice treated with H1N1 influenza virus or antibiotics for 9 days or treated with PBS were analyzed by GC/TOF-MS and then further examined through partial least squares discriminant analysis (PLS-DA) to compare between the three groups. The 563 metabolic features were examined for pathway enrichment analysis using Metaboanalyst software; **(B)** The metabolic pathways significantly affected by influenza virus infection between the control and virus-infected groups. **(C)** The metabolic pathways significantly affected by influenza virus infection and antibiotic treatment between the control and antibiotic-treated groups.

**Figure 5:**
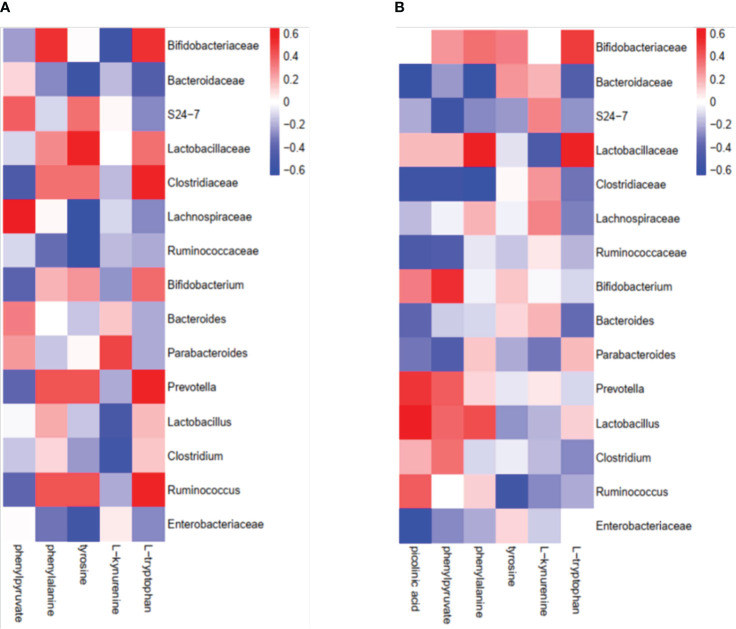
Correlation analysis of intestinal microorganisms and metabolites between the virus-infected group and antibiotic-treated group. **(A)** virus-infected group; **(B)** antibiotic-treatment group.

**Figure 6:**
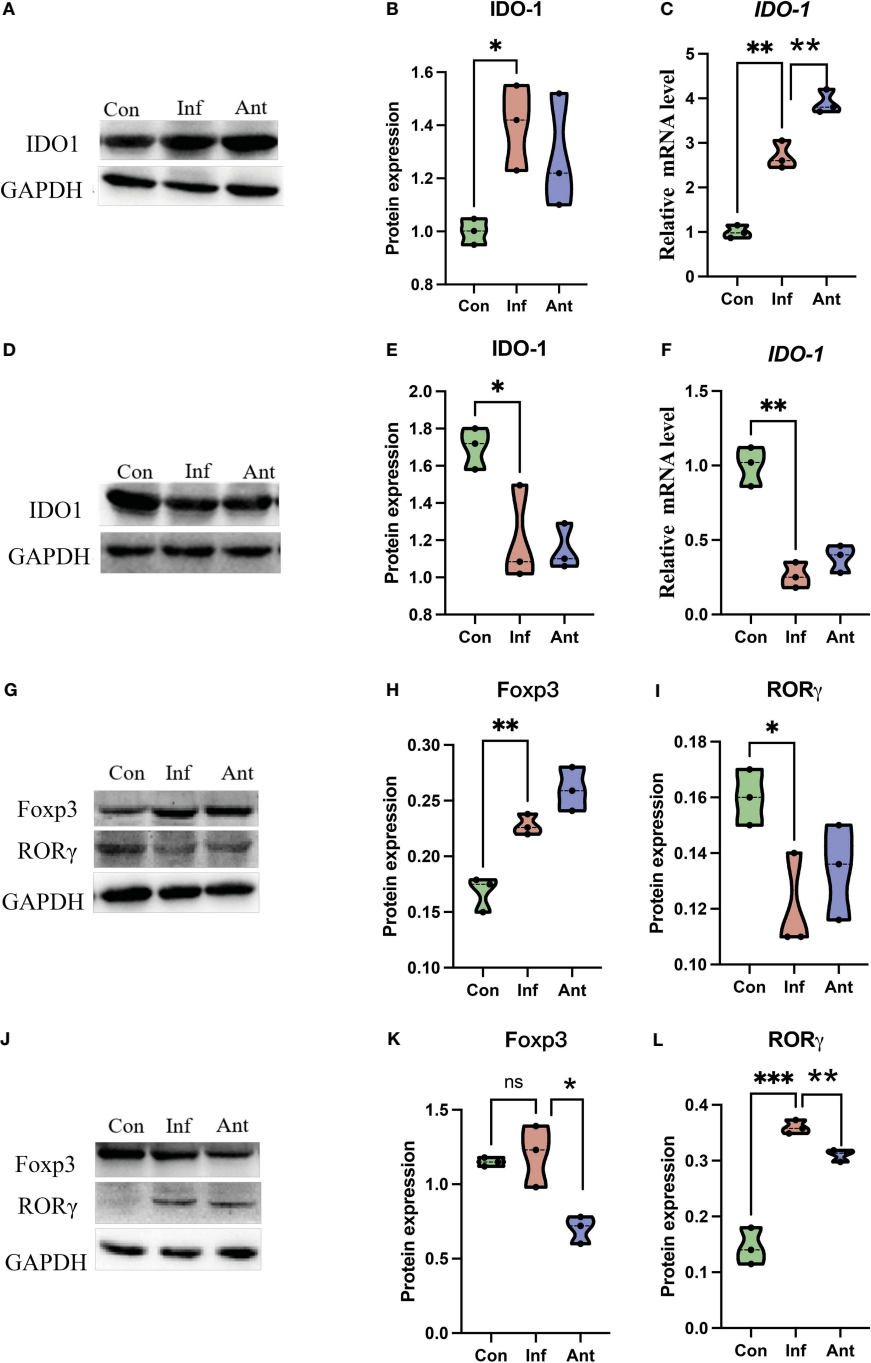
The expression of IDO1, RORγ, and Foxp3 in the colon and ileum. The expression of IDO1 at the transcriptional level and protein level in the **(A–C)** colon and **(D–F)** ileum of the experimental group at the 9th day post infection; RORγ and Foxp3 were detected by immunoblot in the **(G–I)** colon and **(J–L)** ileum of the experimental group at 9th day post infection. Each dot represents data from one animal. Data are mean ± SEM. n = 3. Con: Female BALB/c mice aged 6 to 7 weeks (20 ± 2 g) were treated with PBS; Inf group: Female BALB/c mice aged 6 to 7 weeks (20 ± 2 g) were treated with influenza virus infection; Ant group: Female BALB/c mice aged 6 to 7 weeks (20 ± 2 g) were treated with the combination of antibiotics. Statistical analysis was performed by independent samples T test. *P < 0.05; **P < 0.01; ***P < 0.001; ns, no significant difference. All replicates are biological.

Given that gut bacteria influence host metabolism and immunity through a variety of chemically different metabolites (32), we performed gas chromatography-time of flight mass spectrometry (GC-TOF/MS) to better understand the detailed metabolic information from the virus-infected and antibiotic-treated mice. The data, analyzed by partial least-squares discriminant analysis (PLS-DA), showed significant separation between the virus-infected mice and antibiotic-treated mice, with no obvious differences between virus-infected and mock-infected mice (**Figure 4A**). To determine the effects of intestine microbiota on metabolic processes during the infection, we utilized the Metaboanalyst software to screen the differentially altered metabolic pathways (**Figures 4B, C**). As expected, amino acid metabolism pathways were significantly altered in the feces of the virus-infected and antibiotic-treated mice. Among these pathways, we focused on tryptophan metabolism because correlation analysis revealed a close link with *Lactobacillaceae* and *Enterobacteriaceae* (**Figures 5A, B**), as previously reported (33). Almost all dietary tryptophan is metabolized in mammals by indoleamine 2,3-dioxygenase 1 (IDO1) (34), so we sought to determine if the protein level of IDO1 in the intestines of the virus-infected mice and antibiotic-treated mice was changed. We examined the protein expression of IDO1 in the colon (**Figures 6A–C**) and ileum (**Figures 6D–F**). Surprisingly, at both the transcriptional and protein levels, we found the completely opposite trend of IDO1 between the colon (upregulated) and ileum (downregulated) in both groups. The different expressions of IDO1 in different sites of the intestines suggests that the consequences of the microbiome and metabolomics in fecal contents cannot fully represent the immune condition in the small intestine. Although the microbiota composition and metabolic pathways differed between the virus-infected and antibiotic-treated mice, the trend of IDO1 level was consistent in both groups, indicating that other factors may mediate IDO1 expression, such as the aryl hydrocarbon receptor (AHR) (35) and IFN-γ (36). The level of IDO1 expression in intestinal mucosa indicates that IDO1 is a critical enzyme regulating the local tryptophan state; both dysregulation and overactivation can lead to alterations in immune activation. Thus, in this study, we investigated the tryptophan-influenced immune balance by exploring the equilibrium of Th17 and Treg cells in the colon and ileum of the virus-infected and antibiotic-treated mice. As the specific transcription factor for Th17 and Treg cells, the expression of Retinoic acid-related orphan receptor gamma (RORγ) and Forkhead box p3 (Foxp3) were detected in tissues (**Figures 6G–L**). We found that the expressions of Foxp3 increased and the expression of RORγ decreased in the colon of the experimental groups (**Figures 6G–I**), which contrasts with the findings in the ileum (**Figures 6J–L**). The expression of Foxp3 in the ileum showed no difference between the virus-infected mice and mock-infected mice, which may explain why intestine destruction was much more serious in the antibiotic-treated mice.

**Figure 7:**
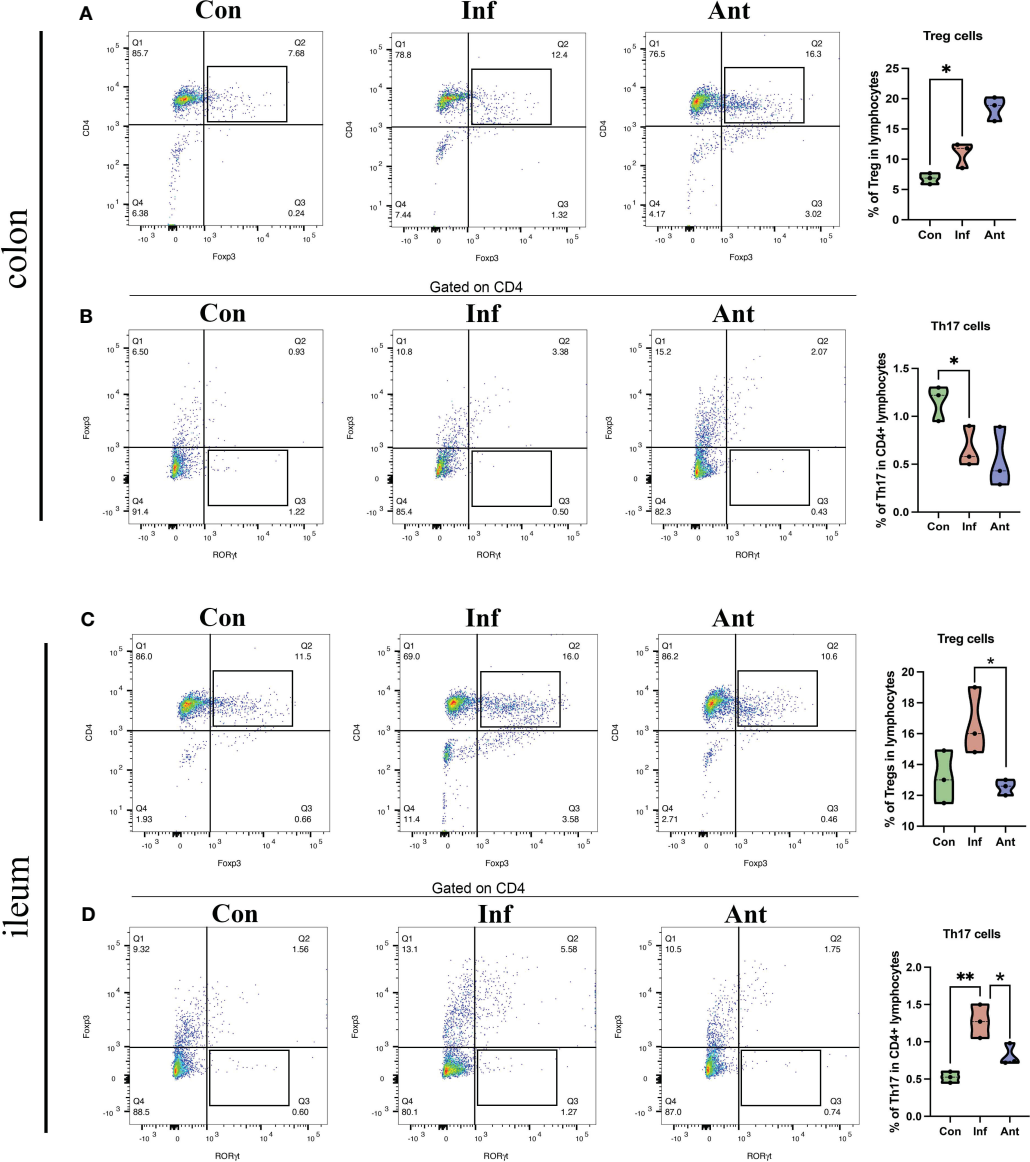
Effects of influenza virus infection on the quantity of Treg cells and Th17 cells in the colon and ileum of mice. The frequencies of Treg cells **(A)**, and Th17 cells **(B)** of colon lymphocytes were analyzed by flow cytometry. The frequencies of Treg cell **(C)**, and Th17 cells **(D)** of ileum lymphocytes were analyzed by flow cytometry. Treg cell, CD4+Foxp3+; Th17 cell, CD4+Foxp3-RORγt+. Each dot represents data from one animal. Data are mean ± SEM. n = 3. Con: Female BALB/c mice aged 6 to 7 weeks (20 ± 2 g) were treated with PBS; Inf group: Female BALB/c mice aged 6 to 7 weeks (20 ± 2 g) were treated with influenza virus infection; Ant group: Female BALB/c mice aged 6 to 7 weeks (20 ± 2 g) were treated with combination of antibiotics. Statistical analysis was performed by independent samples T test. *P < 0.05; **P < 0.01. All replicates are biological.

Additionally, we isolated lymphocytes from the colon and ileum and used flow cytometry to separate T cells in the intestine, further examining the quantities of Treg cells (CD4+Foxp3+) and Th17 cells (CD4+Foxp3-RORγt+). The results are shown in **Figure 7**. Influenza virus infection significantly increased the number of Treg cells in the colon (**Figure 7A**) and decreased the number of Th17 cells (**Figure 7B**). Furthermore, the influenza virus did not significantly affect the number of Treg cells in the ileum (**Figure 7C**), but there was a significant increase in the number of Th17 cells in the ileum following influenza virus infection (**Figure 7D**).

### 3.5 Regulatory effects of tryptophan and *Lactobacillus* in mice with influenza virus

**Figure 8:**
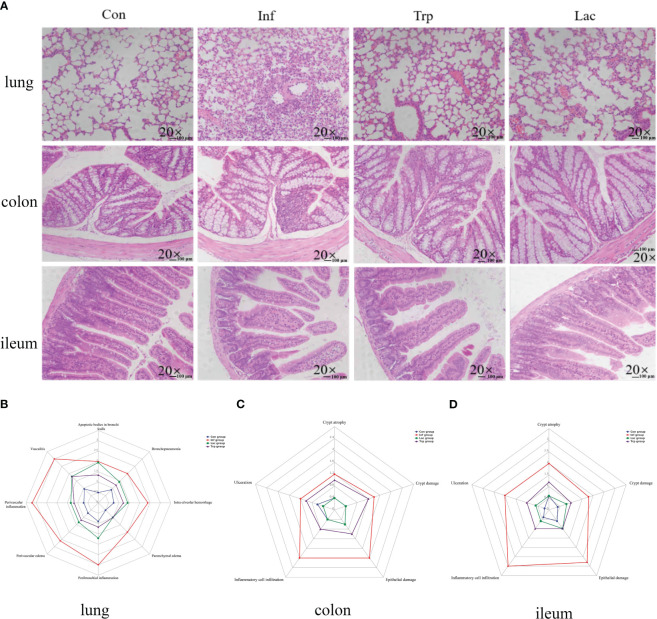
Effects of the influenza virus, tryptophann and Lactobacillus on lung and intestinal tissue structure. **(A)** Histology analysis including H&E (scale: 100 μm) of the lungs, colon, and ileum was observed at 9 days post infection; **(B)** Spider web plot displaying histopathological scoring of lung damage; **(C)** Spider web plot displaying histopathological scoring of colon damage; **(D)** Spider web plot displaying histopathological scoring of ileum damage. Con: Female BALB/c mice aged 6 to 7 weeks (20 ± 2 g) were treated with PBS; Inf group: Female BALB/c mice aged 6 to 7 weeks (20 ± 2 g) were mice treated with influenza virus infection; Trp group: Female BALB/c mice aged 6 to 7 weeks (20 ± 2 g) were treated with exogenous tryptophan supplement; Lac group: Female BALB/c mice aged 6 to 7 weeks (20 ± 2 g) were treated with exogenous Lactobacillus supplement.

This study investigated the role of microorganisms and metabolites in the damage caused by the influenza virus in lung and intestinal tissues through supplementation of *Lactobacillus* and tryptophan in the gastrointestinal tract during influenza infection. As shown in **Figures 8A, B**, lung tissue sections in the control group showed integrity alveolar morphology, no secretions in the cavity, and no inflammatory cell infiltration. While in the virus-infected group, the alveolar structure was disordered and there was a large number of inflammatory cell infiltrations. Alveolar morphology in the tryptophan group and *Lactobacillus* group was similar, with a small number of inflammatory cell infiltrations. The colon and ileum sections of the control group showed complete epithelial structure and uniform villi length, while the colon and ileum were damaged in the virus-infection group with inflammatory cell infiltration and epithelial damage (**Figures 8C, D**). However, the tryptophan group and the *Lactobacillus* supplement group significantly alleviated lung and intestinal damage caused by influenza virus infection. This was evident in reduced peribronchial inflammation, ameliorated crypt damage, crypt atrophy, and inflammatory cell infiltration in the colon and ileum.

**Figure 9:**
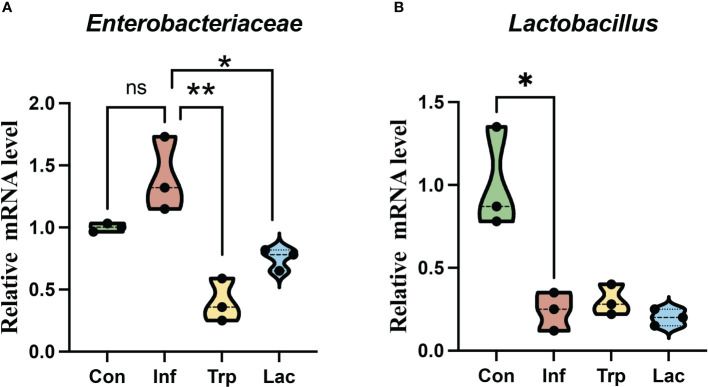
Effects of influenza virus, tryptophan and Lactobacillus supplement on the expression of **(A)** Enterobacteriaceae and **(B)** Lactobacillus in the intestine. Each dot represents data from one animal. Data are mean ± SEM. n = 3. Con: Female BALB/c mice aged 6 to 7 weeks (20 ± 2 g) were treated with PBS; Inf group: Female BALB/c mice aged 6 to 7 weeks (20 ± 2 g) were mice treated with influenza virus infection; Trp group: Female BALB/c mice aged 6 to 7 weeks (20 ± 2 g) were treated with exogenous tryptophan supplement; Lac group: Female BALB/c mice aged 6 to 7 weeks (20 ± 2 g) were treated with exogenous Lactobacillus supplement. Statistical analysis was performed by independent samples T test. *P < 0.05; **P < 0.01. All replicates are biological.

The expression of *Enterobacteriaceae* (**Figure 9A**) and *Lactobacillus* (**Figure 9B**) in the intestines was detected by qPCR. Compared with the control group, the number of *Enterobacteriaceae* increased in the infected group with no significant difference. Compared with the infection group, the number of *Enterobacteriaceae* was significantly reduced in the tryptophan group and *Lactobacillus* group. Compared with the control group, the number of *Lactobacillaceae* in the infected group was significantly reduced. Compared with the infection group, there was no significant difference between the tryptophan group and the *Lactobacillus* group.

**Figure 10:**
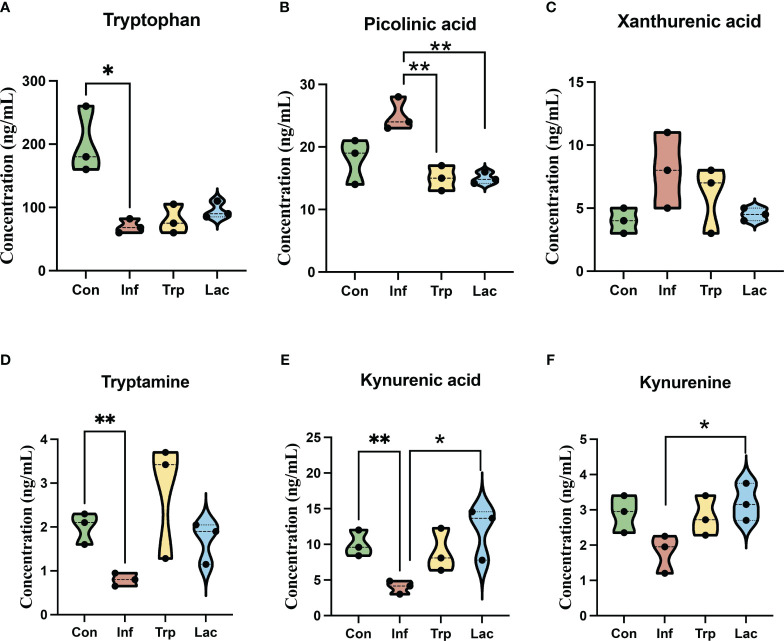
Effects of influenza virus infection and exogenous supplementation of tryptophan and Lactobacillus on the concentration of tryptophan-related metabolites in the intestine of mice. The concentration of **(A)** Tryptophan; **(B)** Picolinic acid; **(C)** Xanthurenic acid; **(D)** Tryptamine; **(E)** Kynurenic acid; **(F)** Kynurenine in the Con, Inf, Try, and Lac groups at 9th day post infection in the cecum microbiota. Each dot represents data from one animal. Data are mean ± SEM. n = 3. Con: Female BALB/c mice aged 6 to 7 weeks (20 ± 2 g) were treated with PBS; Inf group: Female BALB/c mice aged 6 to 7 weeks (20 ± 2 g) were mice treated with influenza virus infection; Trp group: Female BALB/c mice aged 6 to 7 weeks (20 ± 2 g) were treated with exogenous tryptophan supplement; Lac group: Female BALB/c mice aged 6 to 7 weeks (20 ± 2 g) were treated with exogenous Lactobacillus supplement. Statistical analysis was performed by independent samples T test. *P < 0.05; **P < 0.01. All replicates are biological.

We performed liquid chromatography-mass spectrometry (LC-MS) to analyze the concentration of metabolites in the tryptophan metabolic pathway (**Figure 10**). The concentration of tryptophan was not significantly different between the control and infection groups and the concentration of tryptophan in the tryptophan group and the *Lactobacillus* group showed no significant difference from the infection group (**Figure 10A**). The concentration of picolinic acid in the infected group was not significantly different from that in the control group, and the concentration of picolinic acid in the tryptophan group and *Lactobacillus* group was significantly lower than that in the infected group (**Figure 10B**). Compared with the control group, the concentration of tryptamine (**Figure 10D**), kynurenic acid (**Figure 10E**), and kynurenine (**Figure 10F**) in the infected group was significantly reduced and remarkably increased in the tryptophan group and the *Lactobacillus* group. However, there was no significant difference in the Xanthurenic acid concentration of the four groups (**Figure 10C**).

**Figure 11:**
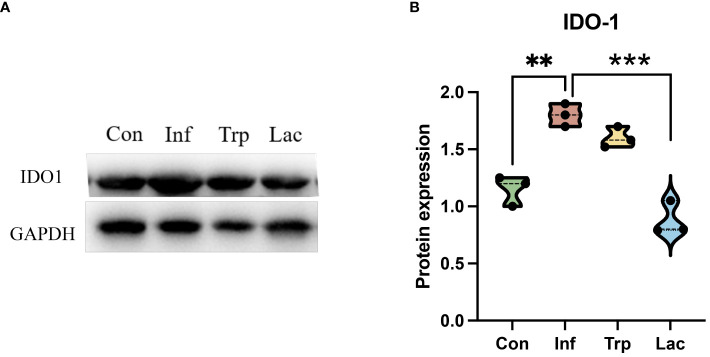
The expression of IDO1 in the intestine. **(A)** Western blot results for IDO1; **(B)** Statistical results of IDO1 expression levels in four groups. Protein expression = IDO1 protein level / GAPDH protein level. Each dot represents data from one animal. Data are mean ± SEM. n = 3. Con: Female BALB/c mice aged 6 to 7 weeks (20 ± 2 g) were treated with PBS; Inf group: Female BALB/c mice aged 6 to 7 weeks (20 ± 2 g) were mice treated with influenza virus infection; Trp group: Female BALB/c mice aged 6 to 7 weeks (20 ± 2 g) were treated with exogenous tryptophan supplement; Lac group: Female BALB/c mice aged 6 to 7 weeks (20 ± 2 g) were treated with exogenous Lactobacillus supplement. Statistical analysis was performed by independent samples T test. **P < 0.01; ***P < 0.001. All replicates are biological.

To further evaluate the alteration of metabolites in the tryptophan metabolic pathway, the expression of the rate-limiting enzyme IDO1 in tryptophan metabolism was detected (**Figures 11A, B**). Compared with the control group, the expression of IDO1 in the infection group was significantly increased, the expression of IDO1 in the *Lactobacillus* group was significantly decreased compared with the infection group (P<0.01), and there was no significant change in the tryptophan group.

## 4 Discussion

Influenza virus infection also causes immune injury in the mucosal tissues, implying that an underlying association existed between respiratory and intestinal tract. Thus far, studies have provided evidences to support the concept of “common mucosal immune system” (37). Soner Yildiz and colleagues observed that influenza virus infection had little effect on lower respiratory tract microbiota abundance and composition (38), which indicated that changes in intestine tract microbiota rather than in respiratory tract microbiota are involved in the connection between the lungs and gut after IAV infection. Multiple studies have identified that germ-free mice exhibit impaired gastrointestinal tract (GI) development (39) and an undeveloped adaptive immune system (40) which highlights the importance of the GI microbiota to host immunity. Alterations to the intestinal microbiota not only affect the host’s susceptibility to opportunistic pathogens but also can have a broad impact on the immune status and function of the host.

As a crucial bridge between innate and adaptive immunity, toll-like receptors activate the MyD88-dependent signaling pathway (41), leading to the release of inflammatory mediators such as IFN-β. In our study, we found that the expression of TLR7 signaling and IFN-β were significantly upregulated in lungs attacked by influenza virus alone or with the administration of combinatorial antibiotics. Nevertheless, in the antibiotic-treated mice, the expression of components of the TLR7 signaling pathway was inconsistent with the results reported by others (31). This could be due to the difference in time point for antibiotic administration. In our model, the mice were treated with antibiotics 1 day after infection, while in others, antibiotics were taken at least 1 week before infection with the flu virus (42, 43). We assume that the antibiotic-induced dysbacteriosis in others was worse than that in our model which resulted in the differential expression of TLR7 signaling. Being the downstream signaling-related inflammatory cytokines of TLR7, IFN-β expression was also elevated in the lungs of the virus-infected and antibiotic-treated mice. It has been confirmed that influenza pulmonary infection can significantly alter the intestinal microbiota profile through a mechanism dependent on type I interferon using *Ifnar1* ^–/–^mice, but it is not clear whether one or both of them make the major contribution to this mechanism, because the type I interferon family consists of IFN-α and IFN-β. In our study, IFN-α did not react to the influenza infection, therefore IFN-β may be the key factor in the type I interferon family to influence the intestinal microbiota or the high level of IFN-α did not last for a long time. Interestingly, as shown in the present study, microbiota abundance and composition did not significantly change in the fecal contents of the virus-infected mice in spite of significantly increased IFN-β. One explanation may relate to the variation in immune status in the virus-infected mice at 9 dpi. At this time point, the mice may have been at the stage of self-healing, the recovery of intestinal microorganisms prior to virus clearance and mucosal epithelium repair, and then the use of antibiotics disrupted the microbiota homeostasis.

It is well known that the dynamic interaction between the host and its microbiota have a profound effect on achieving and maintaining homeostasis (44). Our study, coupled with other recent reports, focused on the bacterial species and specific microbial metabolites to better understand the crosstalk between host immunity and microbial mutualism. Colonization of mice by *Clostridium* strains in the colon (45) or *Segmented filamentous bacterium* in the small intestine (46) resulted in resistance to colitis and reduced growth of intestinal pathogens. Treating mice with sensitive drugs for *Escherichia coli* or supplementing with extrinsic probiotics will help to prevent dysbacteriosis and protect mice against immune injury induced by influenza infection. This research confirmed the importance of the manipulation of commensal bacteria in the intestines. The microbiome results in our study revealed increased *Enterobacteriaceae* in the antibiotic-treated mice and decreased *Lactobacillaceae* in both groups. Therefore, the alteration of the opportunistic pathogen—*Enterobacteriaceae* and probiotic—*Lactobacillaceae* in the intestine will influence host immunity.

There was a significant change in the tryptophan metabolism pathway in the stool samples of the antibiotic-treated mice in our study. Tryptophan metabolism can be divided into endogenous host metabolites and microbiome-modulated metabolites. Since the microbiota can directly and indirectly modulate host endogenous tryptophan metabolism and tryptophan is primarily metabolized through the kynurenine pathway, we detected the expression of IDO1, the rate-limiting enzyme of kynurenine pathway (47), in the colon and small intestine of the virus-infected and antibiotic-treated mice.

The IDO1 expression in the colon was totally opposite to its expression in the small intestine of two groups which may have been caused by the different distribution of gut microbes in different sites. The colon is the home to the most dense and metabolically active community (48) whereas the upper two-thirds of the small intestine only contains low numbers of microorganisms, which suggests that the level of IDO1 may be related to the diversity of gut microbes. Increased IDO1 activity shows immunosuppression and decreased IDO1 activity shows weak resistance to pathogens, thus a delicate balance between pathogen defense and host protection is important to keep immune homeostasis in the intestine. This double-edged sword function of IDO1 explains why the different expression of IDO1 in both the colon and small intestine could cause injury.

In addition to being a rate-limited enzyme in the kynurenine pathway, IDO1 also plays an essential role in maintaining an immune environment. The depletion of tryptophan and production of associated metabolites leads to cell cycle arrest of immune cells, activates regulatory T-cells and blocks their conversion into Th17-like cells (49). In this study, the expression of Foxp3 and RORγ in the colon and small intestine was basically consistent with the immunoregulatory mechanism of IDO1. Together with the intestinal pathology, it showed that intestinal immune damage was related to Th17/Treg imbalance, and Th17/Treg was a good indicator to evaluate intestinal damage. Combining histology and protein expression, the intestines of the antibiotic group had more serious injuries and an increase of *Enterobacteriaceae* was only detected in the antibiotic group. These data might confirm our hypothesis that the increase of *Enterobacteriaceae* might be the main cause of intestinal immune injury during influenza infection which was in accord with an earlier report.

Our study showed that *Lactobacillus* and tryptophan in the intestinal tract were significantly reduced after influenza infection. The changes of microorganisms and metabolites caused serious damage to the respiratory tract and intestinal tract of the hosts. Oral probiotics not only have a direct effect on the intestinal tract, but also have immunomodulatory effects on parenteral tissues, such as the improvement of inflammatory bowel disease and pulmonary infection. Tryptophan is not only an essential nutrient for the body, but plays a vital role in the maintenance of intestinal immune tolerance and the balance of intestinal microbiota. To investigate the regulatory effects of *Lactobacillus* and tryptophan on influenza virus-induced damage, we analyzed the effects of *Lactobacillus* and tryptophan in the respiratory tract and intestinal tract of influenza infected mice. Hematoxylin and eosin (H&E) staining showed that supplementation with both *Lactobacillus* and tryptophan had the remission effect on the damage in the lungs and intestines caused by influenza. *Lactobacillus* can reduce the severity of colitis by improving metabolites in serum and intestinal microbiota, indicating that *Lactobacillus* can prevent tissue damage caused by colitis (50). The lack of dietary tryptophan would impair the intestinal immunity of mice and change the intestinal microbial community, indicating that tryptophan is an important regulatory factor for maintaining intestinal mucosal homeostasis (51). These results are consistent with the effects of *Lactobacillus* and tryptophan on lung and intestinal mucosal injury in this study.

**Figure 12:**
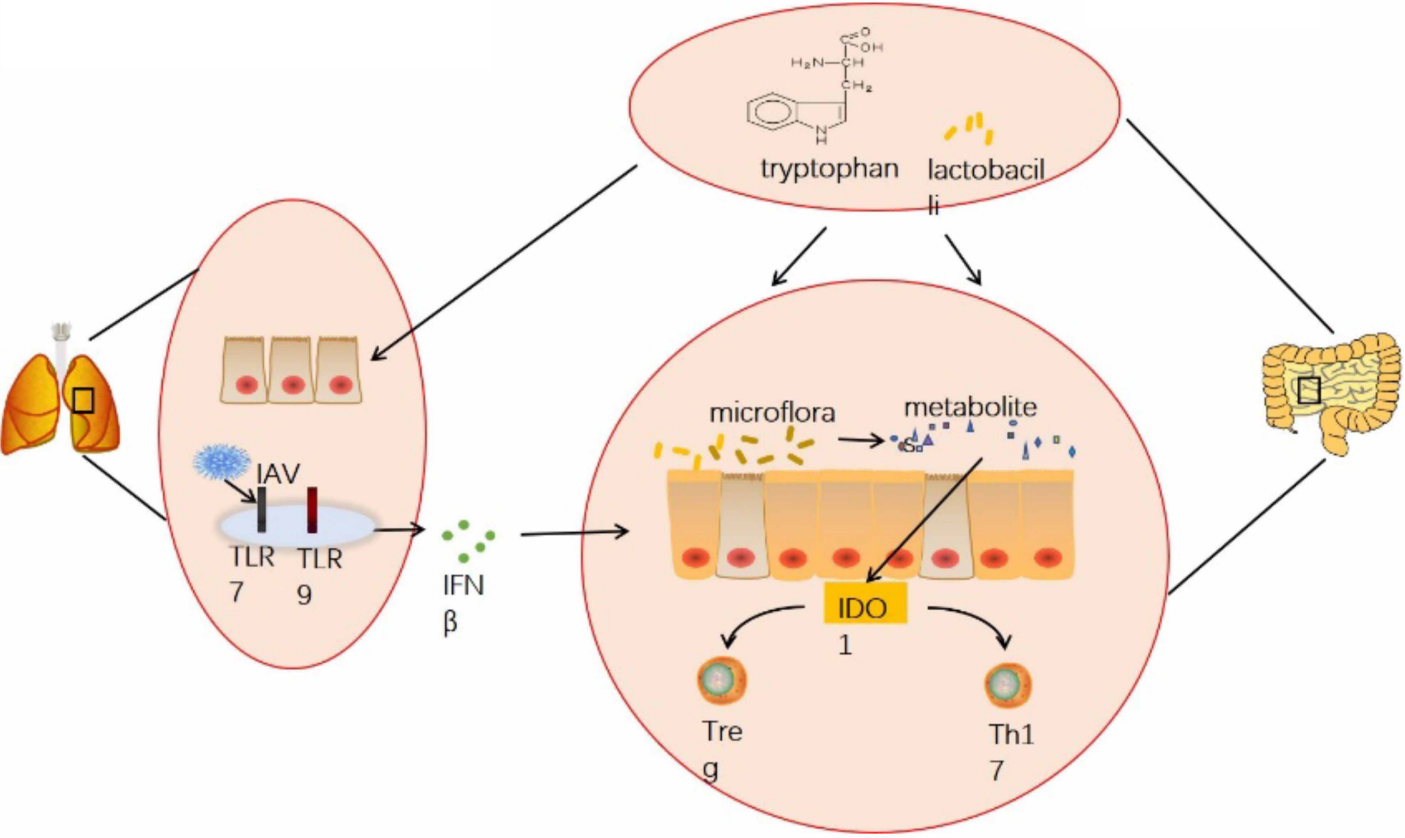
Graphical abstract. Infection with H1N1 influenza virus not only damages the lungs of mice but also causes injury to intestinal epithelial cells and crypt atrophy in the intestine. It induces dysbiosis of the intestinal microbiota, leading to decreased diversity and richness of gut microbiota, reduced abundance of Lactobacillus, and inhibition of metabolic products especially related to tryptophan metabolism pathways. This imbalance results in disrupted proportions of Treg cells and Th17 cells in the intestines, impairing intestinal mucosal immune function. Antibiotic treatment did not significantly improve these effects. However, supplementation with exogenous tryptophan or treatment with Lactobacilli significantly improved pathological damage to the lungs and intestines caused by influenza virus infection, restored the balance of Treg cells and Th17 cells, and recovered intestinal mucosal immune function.

In summary, our study suggested that influenza virus infection can affect the homeostasis of the gut microbiota through the lung-gut axis, thereby influencing the levels of tryptophan-related metabolites in the gut microbiota. Additionally, indoleamine 2,3-dioxygenase 1(IDO1), which acts as a key rate-limiting enzyme in tryptophan metabolism is significantly affected by influenza virus infection. Our experimental results indicated that influenza virus infection can significantly increase the expression of IDO1 in the colon. When IDO1 activity is enhanced, tryptophan consumption accelerates, leading to a decrease in tryptophan concentration. This promotes the production of Tregs and inhibits the helper T cell (Th17) response, thereby suppressing intestinal mucosal immune function. Ultimately, influenza virus infection can cause intestinal pathological damage and hinder intestinal health and development (**Figure 12**). In subsequent experiments, we plan to use IDO1 knockout mice to further investigate the specific mechanisms.

## Data availability statement

The sequencing data we generated were deposited in the NCBI Sequence Read Archive (SRA) under accession numbers from SRR29780069 to SRR29780089 in PRJNA1134695.

## Ethics statement

All animal experiments were conducted under anesthesia in accordance with the Animal Ethics Procedures and Guidelines of the People’s Republic of China and the Institutional Animal Care and Use Committee of Nanjing Agricultural University (SYXK (Su) 2017-0007). The study was conducted in accordance with the local legislation and institutional requirements.

## Author contributions

YZ: Methodology, Investigation, Formal analysis, Data curation, Writing – review & editing, Software. YW: Software, Writing – original draft. XX: Writing – original draft, Formal analysis. YQ: Writing – original draft, Software. WQ: Writing – original draft, Methodology. JP: Resources, Writing – review & editing, Funding acquisition. JS: Resources, Investigation, Data curation, Writing – original draft, Funding acquisition, Formal analysis.

## References

[1] Lathrop SK Bloom SM Rao SM Nutsch K Lio CW Santacruz N. Peripheral education of the immune system by colonic commensal microbiota. Nature. (2011) 478:250–4. doi: 10.1038/nature10434 PMC3192908 21937990

[2] Gray J Oehrle K Worthen G Alenghat T Whitsett J Deshmukh H. Intestinal commensal bacteria mediate lung mucosal immunity and promote resistance of newborn mice to infection. Sci Trans Med. (2017) 9:eaaf9412. doi: 10.1126/scitranslmed.aaf9412 PMC5880204 28179507

[3] Brightbill HD Modlin RL. Toll-like receptors: molecular mechanisms of the mammalian immune response. Immunology. (2000) 101:1–10. doi: 10.1046/j.1365-2567.2000.00093.x 11012747 PMC2327044

[4] Unterholzner L Bowie AG. The interplay between viruses and innate immune signaling: recent insights and therapeutic opportunities. Biochem Pharmacol. (2008) 75:589–602. doi: 10.1016/j.bcp.2007.07.043 17868652

[5] Doyle S Vaidya S O'Connell R Dadgostar H Dempsey P Wu T. Irf3 mediates a Tlr3/Tlr4-specific antiviral gene program. Immunity. (2002) 17:251–63. doi: 10.1016/S1074-7613(02)00390-4 12354379

[6] Shahangian A Chow EK Tian X Kang JR Ghaffari A Liu SY. Type I Ifns mediate development of postinfluenza bacterial pneumonia in mice. J Clin Invest. (2009) 119:1910–20. doi: 10.1172/JCI35412 PMC2701856 19487810

[7] Valdes AM Walter J Segal E Spector TD. Role of the gut microbiota in nutrition and health. BMJ (Clinical Res Ed). (2018) 361:k2179. doi: 10.1136/bmj.k2179 PMC6000740 29899036

[8] Levy M Blacher E Elinav E. Microbiome, metabolites and host immunity. Curr Opin Microbiol. (2017) 35:8–15. doi: 10.1016/j.mib.2016.10.003 27883933

[9] Yu B Dai CQ Chen J Deng L Wu XL Wu S. Dysbiosis of gut microbiota induced the disorder of helper T cells in influenza virus-infected mice. Hum Vaccines Immunother. (2015) 11:1140–6. doi: 10.1080/21645515.2015.1009805 PMC4514154 25874358

[10] Frank DN St Amand AL Feldman RA Boedeker EC Harpaz N Pace NR. Molecular-phylogenetic characterization of microbial community imbalances in human inflammatory bowel diseases. Proc Natl Acad Sci U States A. (2007) 104:13780–5. doi: 10.1073/pnas.0706625104 PMC1959459 17699621

[11] Koh A De Vadder F Kovatcheva-Datchary P Bäckhed F. From dietary fiber to host physiology: short-chain fatty acids as key bacterial metabolites. Cell. (2016) 165:1332–45. doi: 10.1016/j.cell.2016.05.041 27259147

[12] Holmes E Li JV Athanasiou T Ashrafian H Nicholson JK. Understanding the role of gut microbiome-host metabolic signal disruption in health and disease. Trends Microbiol. (2011) 19:349–59. doi: 10.1016/j.tim.2011.05.006 21684749

[13] Qiu J Heller JJ Guo X Chen ZM Fish K Fu YX. The aryl hydrocarbon receptor regulates gut immunity through modulation of innate lymphoid cells. Immunity. (2012) 36:92–104. doi: 10.1016/j.immuni.2011.11.011 22177117 PMC3268875

[14] Round JL Lee SM Li J Tran G Jabri B Chatila TA. The toll-like receptor 2 pathway establishes colonization by a commensal of the human microbiota. Sci (New York NY). (2011) 332:974–7. doi: 10.1126/science.1206095 PMC3164325 21512004

[15] Mazmanian SK Round JL Kasper DL. A microbial symbiosis factor prevents intestinal inflammatory disease. Nature. (2008) 453:620–5. doi: 10.1038/nature07008 18509436

[16] Fallarino F Grohmann U You S McGrath BC Cavener DR Vacca C. The combined effects of tryptophan starvation and tryptophan catabolites down-regulate T cell receptor zeta-chain and induce a regulatory phenotype in naive T cells. J Immunol (Baltimore Md 1950). (2006) 176:6752–61. doi: 10.4049/jimmunol.176.11.6752 16709834

[17] Baban B Chandler PR Sharma MD Pihkala J Koni PA Munn DH. Ido activates regulatory T cells and blocks their conversion into Th17-like T cells. J Immunol (Baltimore Md 1950). (2009) 183:2475–83. doi: 10.4049/jimmunol.0900986 PMC3677163 19635913

[18] Wang X Wang Z Cao J Dong Y Chen Y. Gut microbiota-derived metabolites mediate the neuroprotective effect of melatonin in cognitive impairment induced by sleep deprivation. Microbiome. (2023) 11:17. doi: 10.1186/s40168-022-01452-3 36721179 PMC9887785

[19] Schafer KA Eighmy J Fikes JD Halpern WG Hukkanen RR Long GG. Use of severity grades to characterize histopathologic changes. Toxicol Pathol. (2018) 46:256–65. doi: 10.1177/0192623318761348 29529947

[20] Mann PC Vahle J Keenan CM Baker JF Bradley AE Goodman DG. International harmonization of toxicologic pathology nomenclature: an overview and review of basic principles. Toxicol Pathol. (2012) 40:7s–13s. doi: 10.1177/0192623312438738 22637736

[21] Hogg JC Timens W. The pathology of chronic obstructive pulmonary disease. Annu Rev Pathol. (2009) 4:435–59. doi: 10.1146/annurev.pathol.4.110807.092145 18954287

[22] Dieleman LA Palmen MJ Akol H Bloemena E Peña AS Meuwissen SG. Chronic experimental colitis induced by dextran sulphate sodium (DSS) is characterized by Th1 and Th2 cytokines. Clin Exp Immunol. (1998) 114:385–91. doi: 10.1046/j.1365-2249.1998.00728.x PMC1905133 9844047

[23] Cooper HS Murthy SN Shah RS Sedergran DJ. Clinicopathologic study of dextran sulfate sodium experimental murine colitis. Lab Invest. (1993) 69:238–49. doi: 10.1177/0192623318761348 8350599

[24] Zoetendal EG Akkermans AD De Vos WM. Temperature gradient gel electrophoresis analysis of 16S rRNA from human fecal samples reveals stable and host-specific communities of active bacteria. Appl Environ Microbiol. (1998) 64:3854–9. doi: 10.1128/AEM.64.10.3854-3859.1998 PMC106569 9758810

[25] Wang X Zeng C Lin J Chen T Zhao T Jia Z. Metabonomics approach to assessing the modulatory effects of St John's wort, ginsenosides, and clomipramine in experimental depression. J Proteome Res. (2012) 11:6223–30. doi: 10.1021/pr300891v 23110693

[26] Qiu Y Cai G Zhou B Li D Zhao A Xie G. A distinct metabolic signature of human colorectal cancer with prognostic potential. Clin Cancer Res. (2014) 20:2136–46. doi: 10.1158/1078-0432.CCR-13-1939 PMC5902798 24526730

[27] Fitzgerald KA Kagan JC. Toll-like receptors and the control of immunity. Cell. (2020) 180:1044–66. doi: 10.1016/j.cell.2020.02.041 PMC9358771 32164908

[28] Tian Y Jennings J Gong Y Sang Y. Viral infections and interferons in the development of obesity. Biomolecules. (2019) 9:726. doi: 10.3390/biom9110726 31726661 PMC6920831

[29] Winter SE Thiennimitr P Winter MG Butler BP Huseby DL Crawford RW. Gut inflammation provides a respiratory electron acceptor for salmonella. Nature. (2010) 467:426–9. doi: 10.1038/nature09415 PMC2946174 20864996

[30] Peterson DA Frank DN Pace NR Gordon JI. Metagenomic approaches for defining the pathogenesis of inflammatory bowel diseases. Cell Host Microbe. (2008) 3:417–27. doi: 10.1016/j.chom.2008.05.001 PMC2872787 18541218

[31] Wu S Jiang ZY Sun YF Yu B Chen J Dai CQ. Microbiota regulates the Tlr7 signaling pathway against respiratory tract influenza a virus infection. Curr Microbiol. (2013) 67:414–22. doi: 10.1007/s00284-013-0380-z 23677145

[32] Wikoff WR Anfora AT Liu J Schultz PG Lesley SA Peters EC. Metabolomics analysis reveals large effects of gut microflora on mammalian blood metabolites. Proc Natl Acad Sci USA. (2009) 106:3698–703. doi: 10.1073/pnas.0812874106 PMC2656143 19234110

[33] Zelante T Iannitti RG Cunha C De Luca A Giovannini G Pieraccini G. Tryptophan catabolites from microbiota engage aryl hydrocarbon receptor and balance mucosal reactivity via interleukin-22. Immunity. (2013) 39:372–85. doi: 10.1016/j.immuni.2013.08.003 23973224

[34] Puccetti P Grohmann U. Ido and regulatory T cells: A role for reverse signalling and non-canonical Nf-Kappab activation. Nat Rev Immunol. (2007) 7:817–23. doi: 10.1038/nri2163 17767193

[35] Vogel CF Goth SR Dong B Pessah IN Matsumura F. Aryl hydrocarbon receptor signaling mediates expression of indoleamine 2,3-dioxygenase. Biochem Biophys Res Commun. (2008) 375:331–5. doi: 10.1016/j.bbrc.2008.07.156 PMC2583959 18694728

[36] Campbell BM Charych E Lee AW Möller T. Kynurenines in CNS disease: regulation by inflammatory cytokines. Front Neurosci. (2014) 8:12. doi: 10.3389/fnins.2014.00012 24567701 PMC3915289

[37] Sobko T Schiött J Ehlin A Lundberg J Montgomery S Norman M. Neonatal sepsis, antibiotic therapy and later risk of asthma and allergy. Paediatr Perinatal Epidemiol. (2010) 24:88–92. doi: 10.1111/j.1365-3016.2009.01080.x 20078834

[38] Yildiz S Mazel-Sanchez B Kandasamy M Manicassamy B Schmolke M. Influenza A virus infection impacts systemic microbiota dynamics and causes quantitative enteric dysbiosis. Microbiome. (2018) 6:9. doi: 10.1186/s40168-017-0386-z 29321057 PMC5763955

[39] Hooper LV Littman DR Macpherson AJ. Interactions between the microbiota and the immune system. Sci (New York NY). (2012) 336:1268–73. doi: 10.1126/science.1223490 PMC4420145 22674334

[40] Smith K McCoy KD Macpherson AJ. Use of axenic animals in studying the adaptation of mammals to their commensal intestinal microbiota. Semin Immunol. (2007) 19:59–69. doi: 10.1016/j.smim.2006.10.002 17118672

[41] Barchet W Krug A Cella M Newby C Fischer JA Dzionek A. Dendritic cells respond to influenza virus through TLR7- and PKR-independent pathways. Eur J Immunol. (2005) 35:236–42. doi: 10.1002/eji.200425583 15593126

[42] Ichinohe T Pang IK Kumamoto Y Peaper DR Ho JH Murray TS. Microbiota regulates immune defense against respiratory tract influenza A virus infection. Proc Natl Acad Sci U States America. (2011) 108:5354–9. doi: 10.1073/pnas.1019378108 PMC3069176 21402903

[43] Abt MC Osborne LC Monticelli LA Doering TA Alenghat T Sonnenberg GF. Commensal bacteria calibrate the activation threshold of innate antiviral immunity. Immunity. (2012) 37:158–70. doi: 10.1016/j.immuni.2012.04.011 PMC3679670 22705104

[44] Rooks MG Garrett WS. Gut microbiota, metabolites and host immunity. Nat Rev Immunol. (2016) 16:341–52. doi: 10.1038/nri.2016.42 PMC5541232 27231050

[45] Atarashi K Tanoue T Shima T Imaoka A Kuwahara T Momose Y. Induction of colonic regulatory T cells by indigenous clostridium species. Sci (New York NY). (2011) 331:337–41. doi: 10.1126/science.1198469 PMC3969237 21205640

[46] Ivanov II Atarashi K Manel N Brodie EL Shima T Karaoz U. Induction of intestinal Th17 cells by segmented filamentous bacteria. Cell. (2009) 139:485–98. doi: 10.1016/j.cell.2009.09.033 PMC2796826 19836068

[47] Le Floc'h N Otten W Merlot E. Tryptophan metabolism, from nutrition to potential therapeutic applications. Amino Acids. (2011) 41:1195–205. doi: 10.1007/s00726-010-0752-7 20872026

[48] Sender R Fuchs S Milo R. Are we really vastly outnumbered? Revisiting the ratio of bacterial to host cells in humans. Cell. (2016) 164:337–40. doi: 10.1016/j.cell.2016.01.013 26824647

[49] Andersen MH. The specific targeting of immune regulation: T-cell responses against indoleamine 2,3-dioxygenase. Cancer Immunol Immunother CII. (2012) 61:1289–97. doi: 10.1007/s00262-012-1234-4 PMC3401509 22388712

[50] Vientós-Plotts AI Ericsson AC Rindt H Reinero CR. Oral probiotics alter healthy feline respiratory microbiota. Front Microbiol. (2017) 8:1287. doi: 10.3389/fmicb.2017.01287 28744273 PMC5504723

[51] Hashimoto T Perlot T Rehman A Trichereau J Ishiguro H Paolino M. Ace2 links amino acid malnutrition to microbial ecology and intestinal inflammation. Nature. (2012) 487:477–81. doi: 10.1038/nature11228 PMC7095315 22837003

